# Supplementary material for: Lindqvist@Nanoporous MOF-Based Catalyst for Effective Desulfurization of Fuels
Source: Nanomaterials (Basel). 2022 Aug 22;12(16):2887. doi: 10.3390/nano12162887 (PMC9414597; doi:10.3390/nano12162887)
Supplement: Supplementary file 1 [file nanomaterials-12-02887-s001.zip › nanomaterials-1843518-Supplementary Materials.pdf]

# ELECTRONIC SUPPORTING INFORMATION

# Lindqvist@Nanoporous MOF-Based Catalyst for Effective Desulfurization of Fuels

**Simone Fernandes [1], Daniela Flores [1], Daniel Silva [1], Isabel Santos-Vieira [2], Fátima Mirante [1,*], Carlos M. Granadeiro [1,*] and Salete S. Balula [1,*]**

[1] LAQV/REQUIMTE & Department of Chemistry and Biochemistry, Faculty of Sciences, University of Porto, 4169-007 Porto, Portugal

[2] CICECO—Aveiro Institute of Materials, Department of Chemistry, University of Aveiro, 3810-193 Aveiro, Portugal

* Correspondence: fatima.mirante@fc.up.pt (F.M.); cgranadeiro@fc.up.pt (C.M.G.); sbalula@fc.up.pt (S.S.B.)

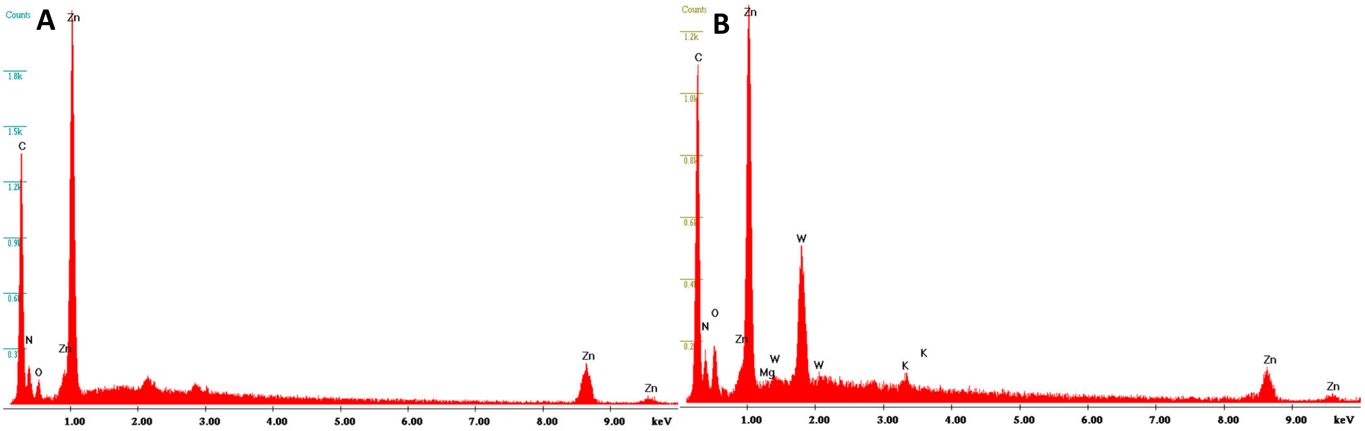


**Figure S1.** EDS spectra of (**A**) ZIF-8 support and (**B**) $EuW_{10}$@ZIF-8 composite.

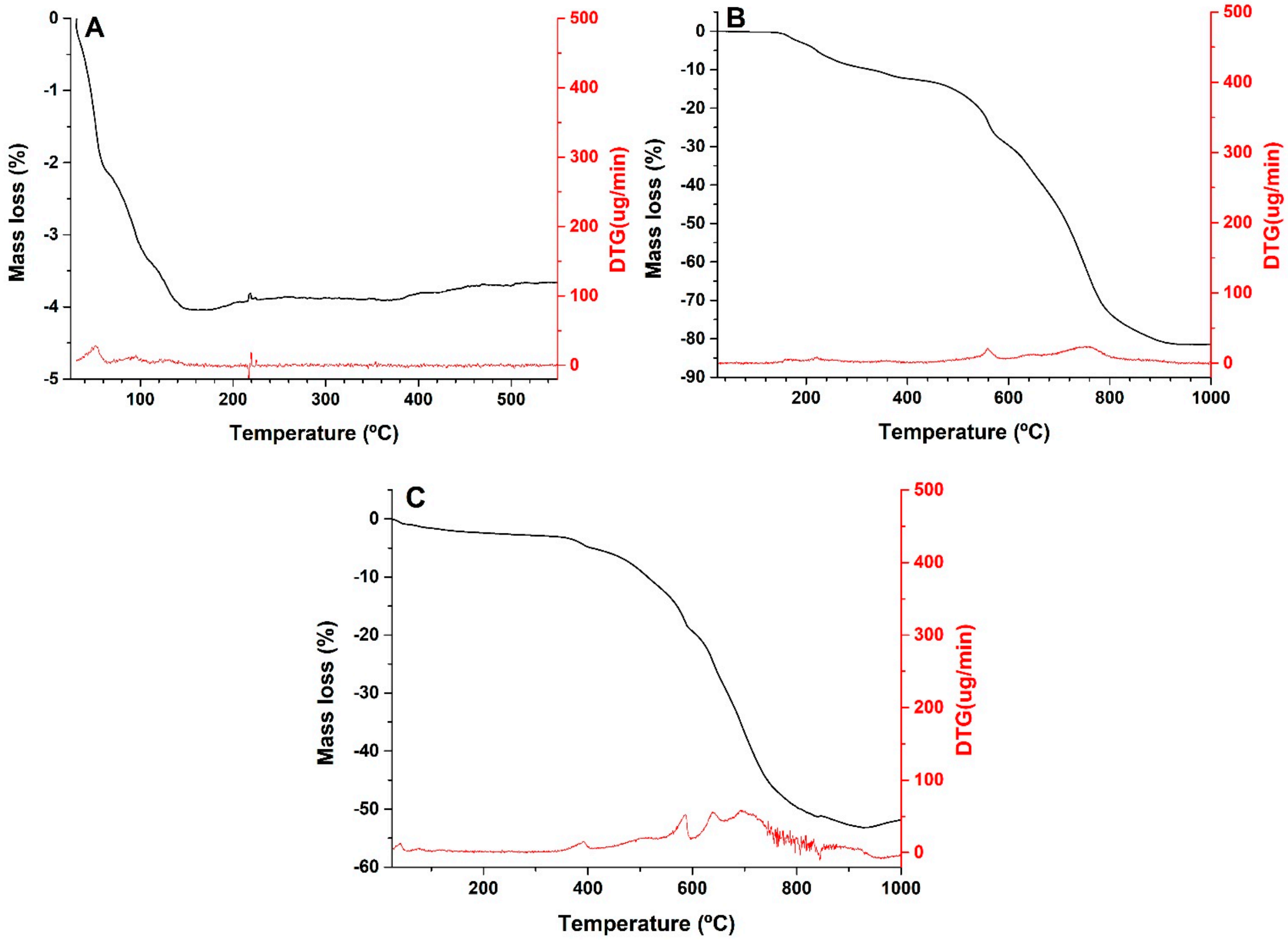


**Figure S2.** Thermogravimetric analysis (TGA) of (A) $EuW_{10}$, (B) ZIF-8 and (C) $EuW_{10}$@ZIF-8 composite.

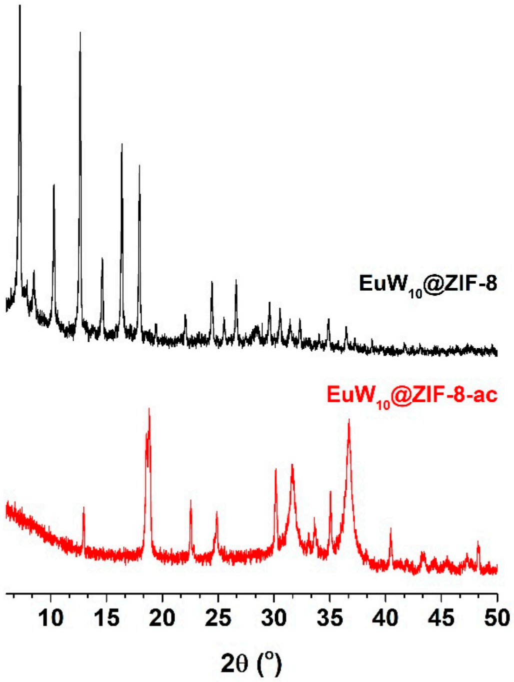


**Figure S3.** Powder XRD patterns of the $EuW_{10}$@ZIF-8 composite as-prepared and after catalysis (ac).

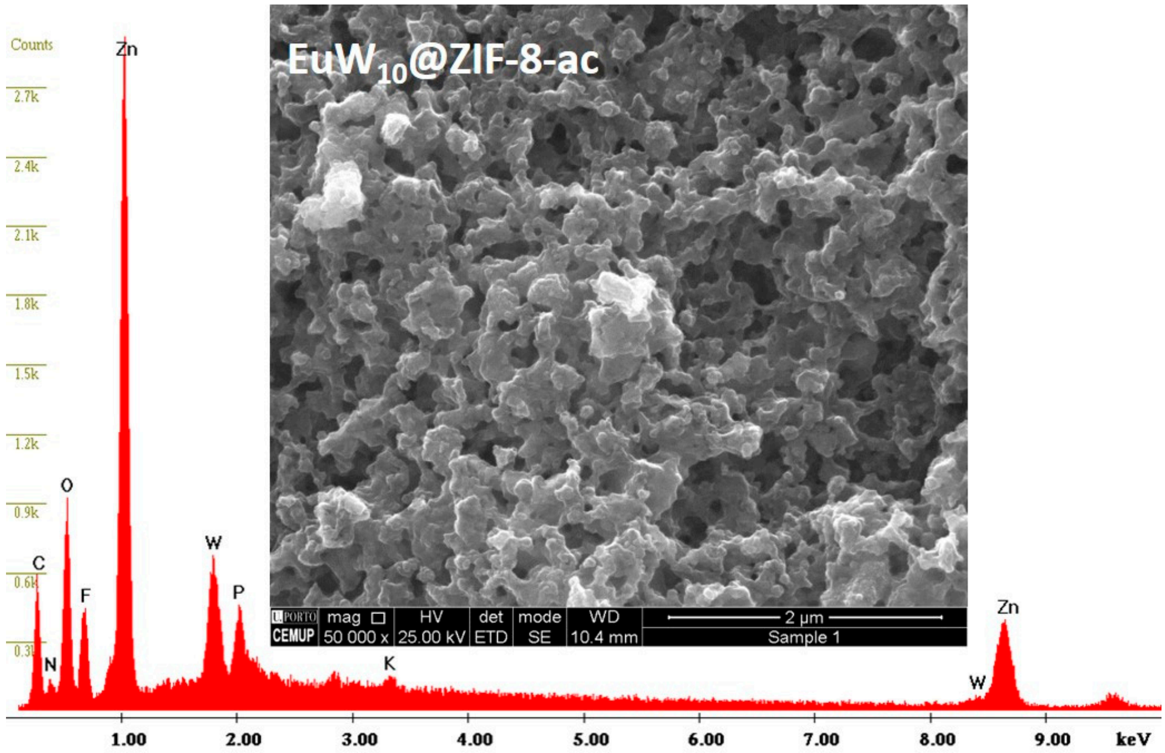


**Figure S4.** SEM image and EDS spectrum of the $EuW_{10}$@ZIF-8 composite after catalysis.
